# Small molecule kinase inhibitor altiratinib inhibits brain cyst forming bradyzoites of *Toxoplasma gondii*

**DOI:** 10.71150/jm.2409001

**Published:** 2025-02-27

**Authors:** Yeong Hoon Kim, Hye-Jin Ahn, Hwa Sun Kim, Ho-Woo Nam

**Affiliations:** 1 Department of Ophthalmology, College of Medicine, The Catholic University of Korea, Seoul 06591, Republic of Korea; 2 Department of Parasitology, College of Medicine, The Catholic University of Korea, Seoul 06591, Republic of Korea; 3 Department of Family Medicine, Veterans Health Service Medical Center, Seoul 05368, Republic of Korea

**Keywords:** *Toxoplasma gondii*, bradyzoite, ME49, small molecule kinase inhibitor, altiratinib

## Abstract

Chronic toxoplasmosis is caused by *Toxoplasma gondii* bradyzoites. This study assessed six candidate small molecule kinase inhibitors (SMKIs) against bradyzoites (ME49 strain), the reactivated form of the parasite resulting from the rupture of brain cysts. Bradyzoites were obtained from mouse brain cysts, cultured in ARPE-19 cells, and treated with afatinib and neratinib (HER2/HER4 inhibitors), ACTB-1003 and regorafenib (VEGFR-2 inhibitors), or altiratinib and foretinib (c-MET inhibitors). The effects on the growth of *T. gondii* were analyzed by western blot and immunofluorescence assay. Changes in the host cells were assessed using markers for cell viability, apoptosis, necrosis, and autophagy. All inhibitors blocked the growth of bradyzoites, although afatinib was less effective. Afatinib enhanced autophagy signals, while ACTB-1003 and neratinib affected mitochondrial biosynthesis and mitophagy. Altiratinib demonstrated an effect against bradyzoites at the lowest concentration with minimal impact on the host cells. It may be effective in blocking the reactivation of brain cysts in immunodeficiency patients caused by bradyzoites.

## Introduction

*Toxoplasma gondii* is an apicomplexan protozoa and a ubiquitous obligate intracellular parasite. While felids are the definitive hosts for this widespread zoonotic pathogen in nature, all other warm-blooded animals, including humans, can serve as intermediate hosts. The average global human seroprevalence rate for this infection is estimated to be 25.7%, although the overall range was determined to be 0.5–87.7%, depending on a wide variety of geographical, economical or environmental factors (Molan et al., 2019). Most acquired infections are mild and tend to become chronic, especially when they affect the central nervous system. However, in its congenital form, they can cause serious complications, such as stillbirth, miscarriage, or severe neurological (McCannel et al., 1996). Toxoplasmic retinochoroiditis is considered one of the most common causes of infective posterior uveitis and a major contributor to visual impairment, particularly in regions with high prevalence of the infection (McCannel et al., 1996). However, there is no consensus on the effectiveness of antibiotics in treating this condition (Pradhan et al., 2016).

*T. gondii* has three infectious stages caused by tachyzoites, bradyzoites and sporozoites (oocyst), which are linked in a complex life cycle (Dubey et al., 1998). Bradyzoites multiply slowly within tissue cysts. These cysts grow and remain intracellular as bradyzoites divide by endodyogeny (Ferguson and Hutchison, 1987). Intact tissue cysts can persist for the life of the host without inducing a significant inflammatory response. Bradyzoites are less susceptible to destruction by proteolytic enzymes compared to tachyzoites (Jacobs et al., 1960). Frenkel suggested that some tissue cysts may rupture in chronically infected animals, but the released bradyzoites are typically destroyed by an immunocompetent host. However, in immunosuppressed animals, the released bradyzoites are believed to reactivate *T. gondii* infection (Frenkel, 1956). Additionally, only the bradyzoite stage reliably produces oocyst excretion in cats (Dubey et al., 1998). These factors emphasize the importance of targeting bradyzoites in the treatment of toxoplasmosis.

Protein kinases (PKs) regulate most cellular pathways, particularly those involved in signal transduction. Among them, protein tyrosine kinases (TKs) play a key role in activating many proteins through phosphorylation. This activation occurs when polypeptide ligands bind to cell surface receptors that have tyrosine kinase catalytic activity (Hubbard and Till, 2000). Numerous TKs have been identified as oncogenes in various tumors and are associated with diseases such as diabetic retinopathy, atherosclerosis, psoriasis (Levitzki and Gazit, 1995), as well as infections (Yang et al., 2016). TKs are crucial for the survival of *T. gondii* in a hostile environment (Muniz-Feliciano et al., 2013). Additionally, structural and functional differences between *T. gondii* and mammalian PKs can be exploited as drug targets (Peixoto et al., 2010). PKs including those of *T. gondii*, can be inhibited by so called ‘small molecule kinase inhibitors (SMKIs)’ that have been developed for chemotherapy in various types of cancer (Zhang et al., 2009), and they have also been used for the treatment of infectious diseases (Lepri et al., 2014; Sun et al., 2014).

In this study, six candidate SMKIs, previously confirmed in blocking the tachyzoite stage (RH strain), were evaluated against the bradyzoite stage (Me49 strain) of *T. gondii*, the reactivated form resulting from a ruptured brain cyst.

## Materials and Methods

### SMKIs and antibodies

SMKIs afatinib (BIBW2992), neratinib (HKI-272), regorafenib (BAY73-4506), altiratinib (DCC-2701), and foretinib (GSK1363089) were purchased from Selleck Chemicals (, USA). ACTB-1003 (B2016) was purchased from BioVision (USA). Dimethyl sulfoxide (DMSO, D2650) and pyrimethamine (SML3579) were purchased from MERCK (USA).

Bovine serum albumin was purchased from Bovogen Biologicals (Australia). BAG1 (marker for bradyzoites) and GRA10 (marker for bradyzoite differentiation, anti-mouse monoclonal) antibodies were produced in our laboratory. Antibodies against COX IV (4850S), phospho-NF-κB (3033S), phospho-mTOR (2971S), and Bcl-xL (2762) were purchased from Cell Signaling Technology (USA). PGC-1a (A12348) and β-Tubulin (A12289) were purchased from ABClonal Inc. (USA). Tom20 (sc-17764) was purchased from Santa Cruz Biotechnology (USA). SQSTM1/p62 (PM045) antibody was purchased from MBL International (USA), and the LC3B antibody (NB 100-2220) was purchased from Novus Biologicals, LLC (USA). MitoTracker^TM^ (M7512), Alexa Fluor 488 goat-anti-mouse IgG (A11029), Alexa Fluor 568 goat-anti-mouse IgG (A11031), anti-rabbit IgG (A11034), Alexa Fluor 568 goat-anti-rabbit IgG (Invitrogen, A11036) were purchased from Invitrogen (USA). PDCD4 (D29C6) XP^®^ anti-rabbit monoclonal antibody was purchased from Cell Signaling Technology.

### Cell line, parasite, and culture

ARPE-19 cells (ATCC^®^ CRL-2302, USA) were maintained in Dulbecco’s Modified Eagle Medium Nutrient Mixture F-12 (DMEM/F12, Invitrogen) containing 2 µM L-glutamine, 100 U/ml penicillin, 100 µg/ml streptomycin, 0.25 µg/ml fungizone and 10% fetal bovine serum (FBS, Gibco Life Technologies, USA). Bradyzoites of ME49 strains (ATCC^®^ 505611) were obtained from the brain following ME49 strain infection in BALB/c mice.

ME49 strain bradyzoites were observed for three days to account for their slow metabolism. For each growth period, ME49 strains were identified using BAG1 markers. For the immunofluorescence assays of ME49 bradyzoites, Alexa Fluor 488 goat-anti-Rabbit IgG (Invitrogen, A11034) was used for green fluorescence, while Tom20 (Santa Cruz, sc-17764) and Alexa Fluor 568 goat-anti-mouse IgG (Invitrogen, A11031) were used for red fluorescence.

### Effectiveness of SMKIs

Three categories of kinase-inhibiting small molecules (HER2/HER4 inhibitors, C-MET inhibitors, and VEGFR-2 inhibitors) have been previously reported to inhibit the RH strain (Kim et al., 2017; Yang et al., 2016). Based on these results, we evaluated whether these inhibitors would have a similar effect on the ME49 strain with respect to host cell viability, through expression, autophagy, apoptosis/necrosis, and cellular ROS assays.

The following stock concentrations of small molecule kinase inhibitors (SMKIs) were used in the study: 50 mM for afatinib, altiratinib, forentinib, ACTB-1003, and regorafenib; 40 mM for pyrimethamine; and 10 mM for neratinib. Pyrimethamine at 5 μM served as the positive control, while DMSO was used as the negative control. The SMKIs were dissolved and diluted to the correct concentrations using DMSO. For the DMSO control, the dilution was prepared based on neratinib, which had the lowest concentration. To achieve a final concentration of 5 µM for neratinib, a 1:2000 dilution is needed, and this dilution was used as the DMSO control.

Anti-*Toxoplasma* activity against the RH strain was previously demonstrated (Fig. S1). Based on these findings, afatinib and neratinib were selected at a minimal concentration of 5 μM for the current study. For ACTB-1003, regorafenib, altiratinib, and foretinib, minimal concentrations against the RH strain, as established in prior experiments, were used to determine appropriate doses for the ME49 strain in subsequent assays (Fig. S2).

Harvested bradyzoites were inoculated into ARPE-19 cells (Kim et al., 2017). Briefly, ARPE-19 cells were plated on 24-well plates (Costar, USA). After 24 h of incubation, the plates were washed once with pre-warmed DPBS and replaced with fresh medium containing 10% FBS. Fresh bradyzoites were then added, followed by further experiments to assess the effects of the SMKIs. The drugs were added at 24 and 48 h post-infection and the cultures were maintained for up to 72 h. A longer culture period was necessary for bradyzoites due to their slow metabolism. Each experiment was performed in triplicate.

### Western blot analysis

ARPE-19 cells were plated in 6-well plates at a density of 2.0 × 10^5^ cells/3.0 ml/well. Fresh bradyzoites were added to the plates at a density of 5.0 × 10^5^ parasites/3.0 ml/well. Two hours after infection, the uninvaded parasites were removed by washing with pre-warmed DPBS, and the culture was replenished with fresh, pre-warmed medium containing 10% FBS. The drugs were added at 24 and 48 h post-infection, and the cultures were maintained for up to 72 h. The wells were then washed with DPBS, and the cells were lysed with 1× Laemmli sample buffer (Bio-Rad, USA). The cell lysates were dissolved using 12–15% SDS-PAGE and transferred to nitrocellulose membranes (Whatman GmbH, Germany) using a mini-protean Tetra system (Bio-Rad). The membranes were incubated with 5% skim milk (Difco Laboratories, USA) in PBS with 0.5% Tween 20 (PBST) for 1 h. After washing with PBST, the membranes were incubated with primary antibodies in PBST with 5% skim milk at room temperature for 1 h (or 4°C overnight). The membranes were then incubated with secondary antibodies (anti-rabbit or anti-mouse IgG-horseradish peroxidase in PBST with 5% skim milk) for 1 h. The signals were detected with an ECL western blot kit (Millipore Corporation, USA) and analyzed with an LAS-4000 system (Fuji Film, Japan). Each experiment was performed in triplicate.

### Immunofluorescence assay

ARPE-19 cells were plated in 24-well plates (Costar) at a density of 0.5 × 10^5^ cells/0.5 ml/well. After 24 h, the plates were washed once with pre-warmed DPBS and replaced with fresh medium containing 10% FBS. Fresh bradyzoites were added to the plates at a density of 1.0 × 10^5^ parasites/0.5 ml/well. The drugs were added at 24 and 48 h post-infection and the cultures were maintained for up to 72 h. After the infection period, uninvaded parasites were removed by washing with pre-warmed DPBS, and the culture was replenished with fresh, pre-warmed medium containing 10% FBS. At the indicated time points post-infection, the cells in different wells were fixed with ice-cold methanol for 5 min. The cells were then incubated with anti-*Toxoplasma* antibodies (BAG1, GRA10) and PDCD4 antibodies, diluted 1:200 in 3% BSA/DPBS, for 1 h. This was followed by incubation with Alexa Fluor 488 goat-anti-Rabbit IgG (Invitrogen, A11034) for green fluorescence, and Tom20 (Santa Cruz, sc-17764) and Alexa Fluor 568 goat-anti-mouse IgG (Invitrogen, A11031) or Alexa Fluor 568 goat-anti-rabbit IgG (Invitrogen, A11036) for red fluorescence, all at a dilution of 1:500. The cells were washed five times with DPBS. The coverslips were mounted on slide glasses using mounting medium with DAPI, and then the cells were analyzed under a LSM900 confocal laser scanning microscope (Zeiss, Germany) and an Axioimager M1 Fluorescence Microscope (Zeiss) at high-power fields (×400). Each experiment was performed in triplicate.

### Apoptosis/Necrosis Assay and Cellular ROS Assay

Apoptosis/Necrosis Assay (ab176749) and Cellular ROS Assay (ab113851), both from Abcam (USA), were performed according to the manufacturer’s instructions. Host cells were treated with drugs 24 h after bradyzoite infection. The cells were then cultured for up to 72 h for the Apoptosis/Necrosis Assay and for 24 h for the Cellular ROS Assay. Briefly, assay buffers containing Apopxin, 7-AAD, and CytoCalcein were added to the pre-treated host cells. The cells were then analyzed using an Axioimager M1 Fluorescence Microscope (Zeiss). Apoptotic cells were identified by green staining in the FITC channel, while necrotic cells were identified by red staining in the Texas Red channel. Reactive oxygen species (ROS) analysis was performed by staining the treated cells with 20 µmol/L of DCFDA at 37°C for 45 min. Signals of oxidized DCF at 485/535 nm Ex/Em were read using a Microplate Reader (BioTek Instruments, USA). Results were expressed as mean ± standard deviation (SD). Statistical analysis was analyzed using GraphPad prism software (Version 7). The data was analyzed using Student’s one-way analysis of variance (ANOVA) with post hoc Tukey’s multiple comparisons test. A value of *P* < 0.05 was considered statistically significant. Each experiment was performed in triplicate.

## Results

### Effectiveness of SMKIs

**Fig. 1.**
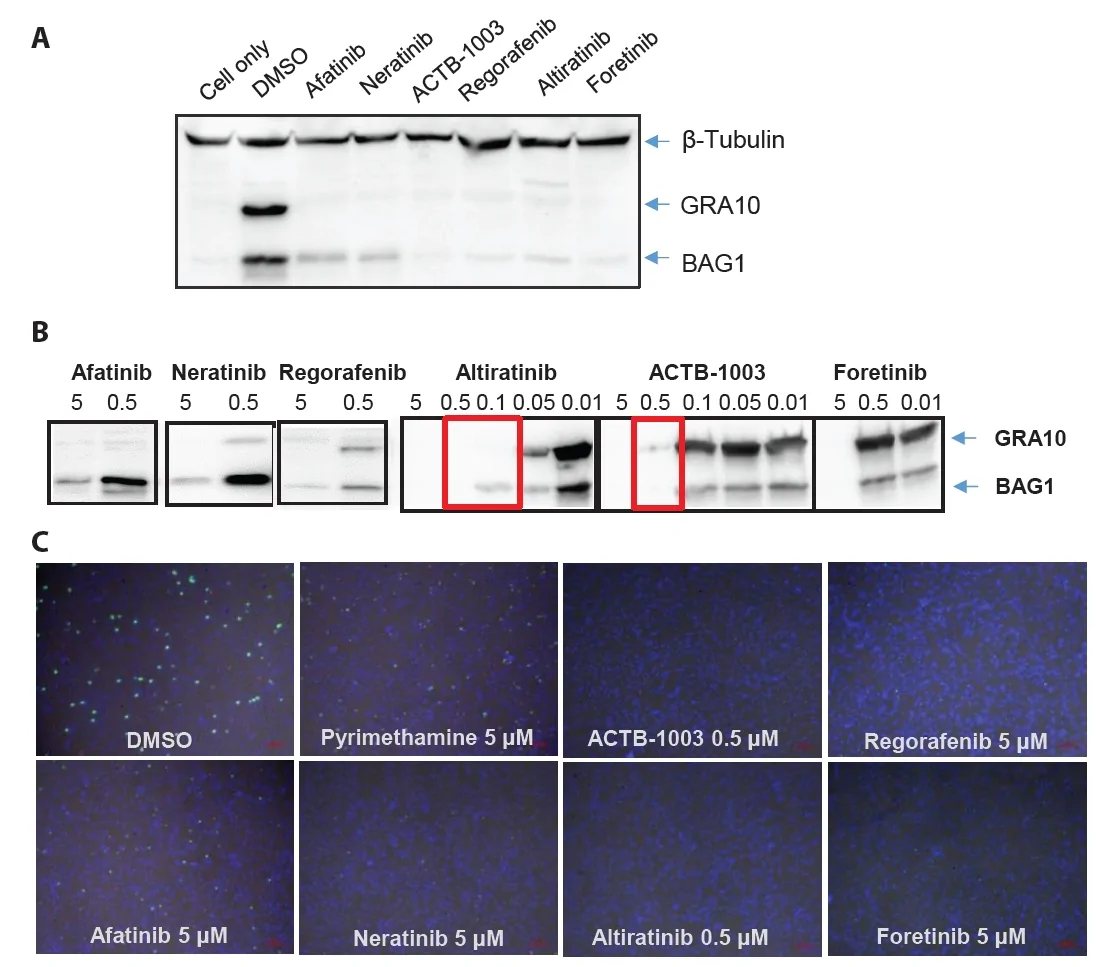
SMKI inhibition of ME49 strain. (A) Western blot analysis of small molecule kinase inhibitors (SMKIs) at 5 μM. At the same concentration, GRA10 expression was inhibited by all SMKIs, while BAG1 expression was only moderately inhibited by afatinib and neratinib. (B) Dose-dependent western blot analysis. ACTB-1003 inhibited both GRA10 and BAG1 at 0.5 μM, whereas altiratinib inhibited GRA10 at 0.1 μM and BAG1 at 0.5 μM. (C) Immunofluorescence assay. SMKIs and pyrimethamine did not significantly affect host cell survival. BAG1 expression was rarely observed in the ACTB-1003 and altiratinib groups at 5 μM. For immunofluorescence assays, GRA10 was detected using Alexa Fluor 488 goat anti-mouse IgG (Invitrogen, A11029) for green fluorescence, and BAG1 was detected using Alexa Fluor 568 goat anti-rabbit IgG (Invitrogen, A11036) for red fluorescence (×100).

Western blot analysis showed that GRA10 expression was significantly decreased by all SMKIs initially at 5 μM in the ME49 strain. BAG1 expression was only moderately reduced by afatinib and neratinib at 5 μM (Fig. 1A). Dose-dependent western blot analysis revealed that ACTB-1003 inhibited both GRA10 and BAG1 at 0.5 μM, while altiratinib inhibited GRA10 and BAG1 at 0.1 and 0.5 μM, respectively.

Based on the above results, altiratinib and ACTB-1003 were used at 0.5 μM, while other SMKIs were used at 5 μM in subsequent experiments (Fig. 1B). The proportions of ARPE-19 cells in the drugs-treated and control groups were analyzed to examine the lethality of the drugs on host cells by immunofluorescence assay. None of the SMKIs or pyrimethamine, used for ME49 strain inhibition, significantly affected the survival of the host cells. BAG1 expression was rarely observed when treated with ACTB-1003 and altiratinib. Regorafenib, a VEGFR inhibitor, showed lower inhibitory activity than ACTB-1003, and foretinib, a C-MET inhibitor, exhibited minimal efficacy compared to altiratinib, leading to their exclusion from further experiments (Fig. 1C).

**Fig. 2.**
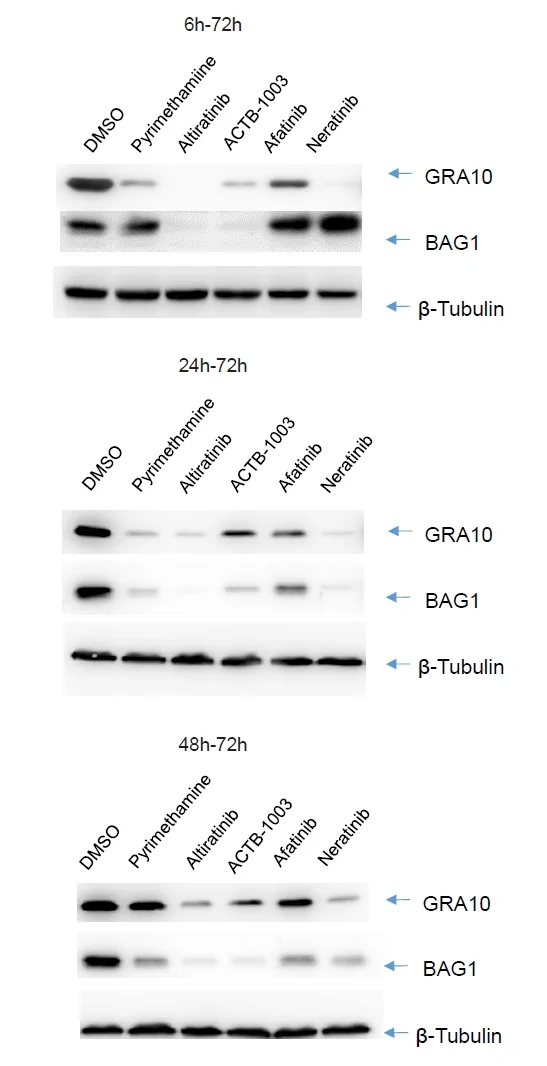
Inhibition of ME49 infection depending on the time of treatment initiation. Altiratinib demonstrated an inhibitory effect at 0.1 μM, while ACTB-1003 exhibited similar effects at 0.5 μM during ME49 infection. In contrast, neratinib increased BAG1 expression during the early stages of ME49 infection. The following concentrations were used: pyrimethamine, afatinib, and neratinib at 5 μM; altiratinib and ACTB-1003 at 0.5 μM (×400).

The expression of BAG1 was evaluated in the treatment groups through western blot analysis, depending on the timing of treatment initiation. BAG1 expression was significantly downregulated with altiratinib at 0.5 μM, regardless of the timing of treatment in the ME49 strain, indicating that altiratinib effectively inhibited bradyzoites. Paradoxically, BAG1 expression increased with neratinib at 5 μM when used during the early stage of infection (Fig. 2).

### Autophagy, apoptosis, necroptosis signals

**Fig. 3.**
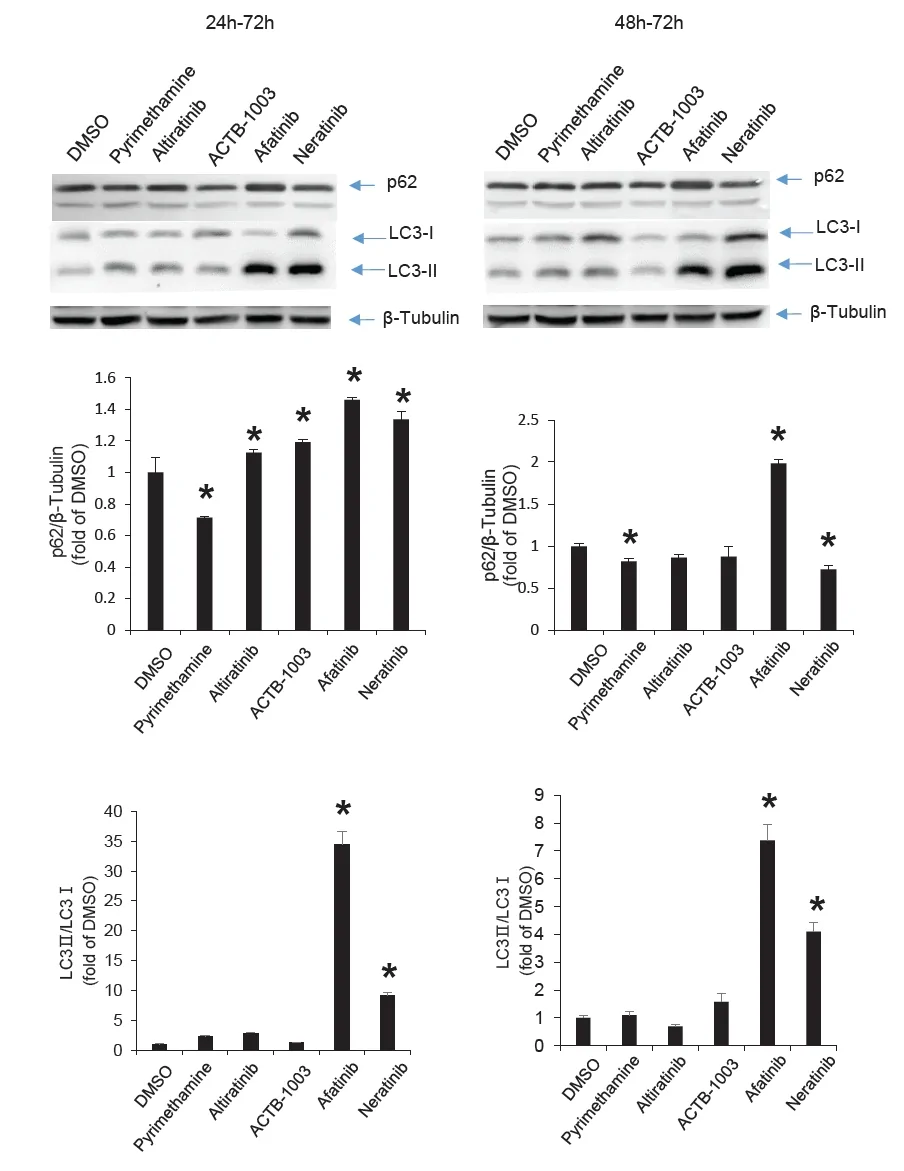
Autophagy. All SMKIs significantly increased p62 expression in the early treatment group (1.13 to 1.46-fold higher than the DMSO control, *P* < 0.05). In contrast, only afatinib at 5 μM showed a significant increase in the late treatment group (1.99-fold higher than the DMSO control, *P* < 0.05). The LC3-II/LC3-I ratio was significantly increased for both afatinib and neratinib at 5 μM. In the early treatment group, the ratios were 34.38-fold and 9.10-fold higher than the DMSO control (*P* < 0.05), respectively. In the late treatment group, the ratios were 7.38-fold and 4.10-fold higher than the DMSO control (*P* < 0.05). On the other hand, although not significant, very low expression levels of p62 and a low LC3-II/LC3-I ratio were observed with altiratinib at 0.5 μM in the late treatment group. The following concentrations were used: pyrimethamine, afatinib, neratinib at 5 μM; altiratinib, ACTB-1003 at 0.5 μM. Asterisks indicate the significant values (^*^ *P* < 0.05).

Autophagy assessment revealed that all SMKIs significantly increased p62 expression in the early treatment group (1.13 to 1.46-fold higher than the DMSO control, *P* < 0.05). In contrast, only afatinib at 5 μM showed a significant increase in the late treatment group (1.99-fold higher than the DMSO control, *P* < 0.05). The LC3-II/LC3-I ratio was significantly increased for both afatinib and neratinib at 5 μM. In the early treatment group, the ratios were 34.38-fold and 9.10-fold higher than the DMSO control (*P* < 0.05), respectively. In the late treatment group, the ratios were 7.38-fold and 4.10-fold higher than the DMSO control (*P* < 0.05). On the other hand, although not significant, very low expression levels of p62 and a low LC3-II/LC3-I ratio were observed with altiratinib at 0.5 μM in the late treatment group (Fig. 3).

**Fig. 4.**
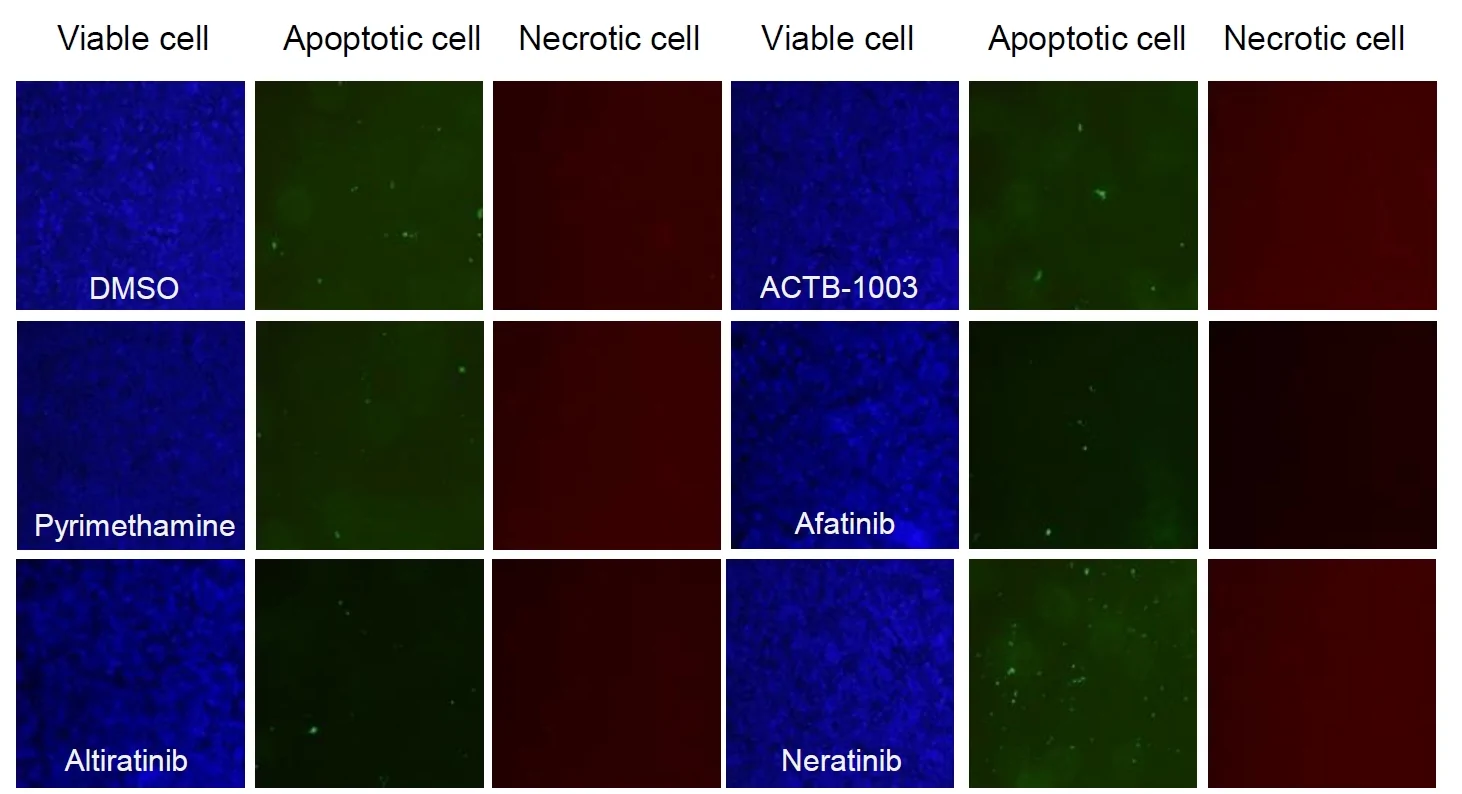
Apoptosis/Necrosis signals. Only a few necrotic cells were observed in all treated groups (afatinib and neratinib at 5 μM; altiratinib and ACTB-1003 at 0.5 μM) as well as in the control groups (DMSO and pyrimethamine at 5 μM). However, a relatively higher number of apoptotic cells were detected with neratinib at 5 μM. Apoptosis/Necrosis analysis was performed using the Apoptosis/Necrosis Assay Kit (Abcam, Ab176749) (×100).

In the apoptosis/necrosis analysis, only a few necrotic cells were observed in all treated groups (afatinib and neratinib at 5 μM; altiratinib and ACTB-1003 at 0.5 μM) as well as in the control groups (DMSO and pyrimethamine at 5 μM). However, a relatively higher number of apoptotic cells were detected with neratinib at 5 μM, leading to its exclusion from further experiments (Fig. 4).

**Fig. 5.**
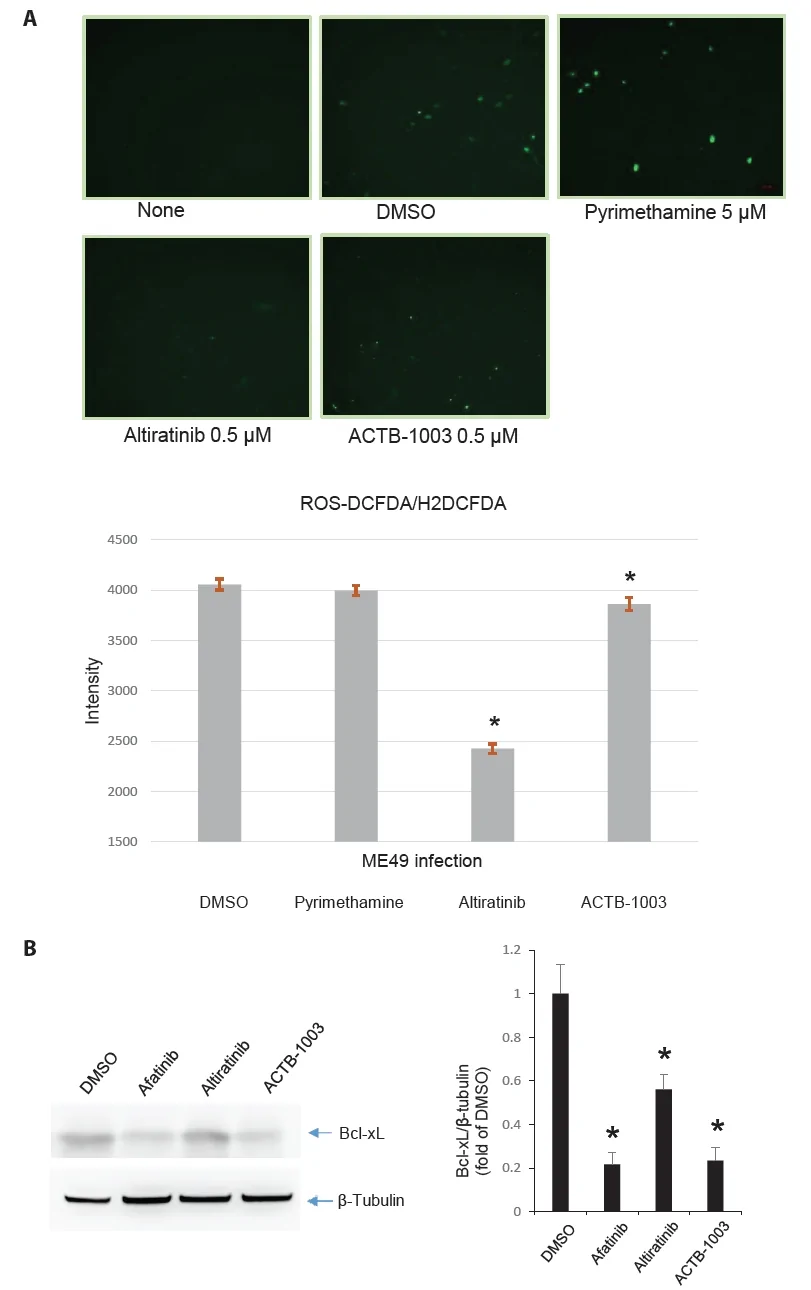
DCFDA/H2DCFDA-Cellular ROS Assay. (A) Treatment of ME49 infection revealed that 2’,7’-dichlorofluorescein (DCF) was more intensely observed with the DMSO control, pyrimethamine at 5 μM, and ACTB-1003 at 0.5 μM. Altiratinib at 0.5 μM resulted in a nearly two-fold significant reduction in ROS intensity compared to the DMSO control (2426 ± 46.64, *P* < 0.05), suggesting that it may have fewer harmful effects on host cells due to ROS during ME49 infection. In contrast, ACTB-1003, while significantly lower than the DMSO control, exhibited relatively higher ROS intensity compared to altiratinib (3861 ± 65.30, *P* < 0.05). (B) Afatinib, altiratinib, and ACTB-1003 all significantly decreased Bcl-xL expression (0.22, 0.56, and 0.23-fold lower than the DMSO control, respectively, *P* < 0.05). However, ACTB-1003 showed lower Bcl-xL expression than altiratinib. Since ACTB-1003 exhibited higher ROS levels and lower Bcl-xL expression than altiratinib, it suggests a potentially more effective induction of apoptosis. The levels of oxidized DCF at 485/535 nm Ex/Em were read using the DCFDA - Cellular ROS Assay Kit / Reactive Oxygen Species Assay Kit (Abcam, ab113851) (×200). Asterisks indicate the significant values (^*^P < 0.05).

Cellular reactive oxygen species (ROS) generation was studied using a fluorescence ROS assay (DCFDA/H2DCFDA) to investigate cell damage caused by oxidative stress from SMKIs. Treatment of ME49 infection revealed that 2’,7’-dichlorofluorescein (DCF), an oxidative product of ROS, was more intensely observed with the DMSO control, pyrimethamine at 5 μM, and ACTB-1003 at 0.5 μM. Altiratinib at 0.5 μM resulted in a nearly two-fold significant reduction in ROS intensity compared to the DMSO control (2426 ± 46.64, *P* < 0.05), suggesting that it may have fewer harmful effects on host cells due to ROS during ME49 infection. In contrast, ACTB-1003, while significantly lower than the DMSO control, exhibited relatively higher ROS intensity compared to altiratinib (3861 ± 65.30, *P* < 0.05) (Fig. 5A). Afatinib, altiratinib, and ACTB-1003 all significantly decreased Bcl-xL expression (0.22, 0.56, and 0.23-fold lower than the DMSO control, respectively, *P* < 0.05). However, ACTB-1003 showed lower Bcl-xL expression than altiratinib. Since ACTB-1003 exhibited higher ROS levels and lower Bcl-xL expression than altiratinib, it suggests a potentially more effective induction of apoptosis. Therefore, ACTB-1003 was excluded from further experiments (Fig. 5B).

**Fig. 6.**
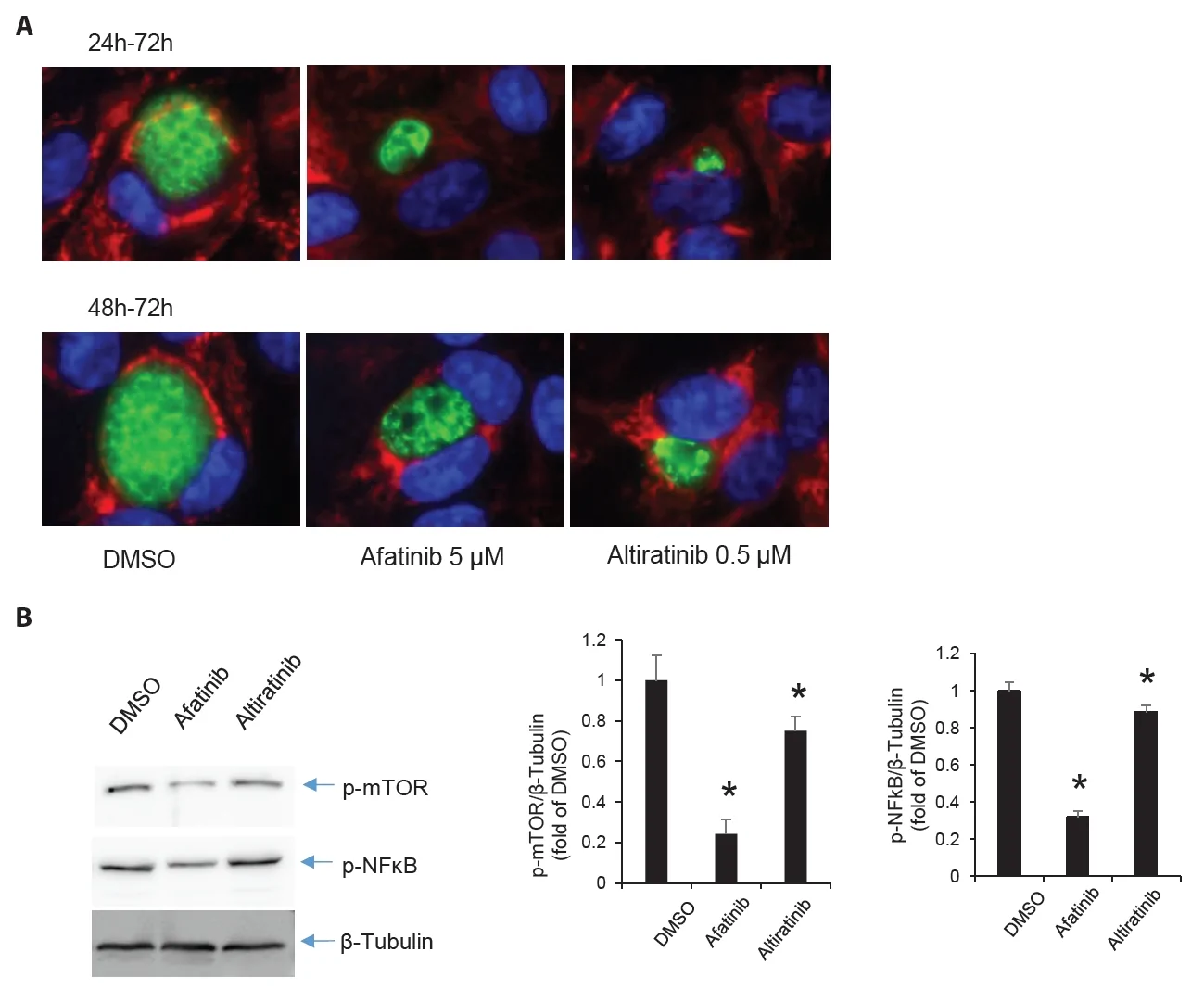
Altiratinib inhibits bradyzoites of *Toxoplasma gondii*. (A) Altiratinib disrupted the intravacuolar network (IVN) within the parasitophorous vacuole (PV) to a much greater extent than afatinib, at both early and late stages of ME49 infection. (B) The expression of phosphorylated mTOR and NF-κB, key regulators of cell growth and immune response, was significantly lower than the DMSO control for both afatinib and altiratinib. However, altiratinib showed relatively higher mTOR (0.24-fold lower than the DMSO control) expression compared to afatinib (0.75-fold lower than the DMSO control). Similarly, altiratinib showed relatively higher NF-κB (0.32-fold lower than the DMSO control) expression compared to afatinib (0.88-fold lower than the DMSO control) (×400). Asterisks indicate the significant values (^*^P < 0.05).

Altiratinib disrupted the intravacuolar network (IVN) within the parasitophorous vacuole (PV) to a much greater extent than afatinib, at both early and late stages of ME49 infection (Fig. 6A). The expression of phosphorylated mTOR and NF-κB, key regulators of cell growth and immune response, was significantly lower than DMSO for both afatinib and altiratinib. However, altiratinib showed relatively higher mTOR (0.24-fold lower than the DMSO control) expression compared to afatinib (0.75-fold lower than the DMSO control). Similarly, altiratinib showed relatively higher NF-κB (0.32-fold lower than the DMSO control) expression compared to afatinib (0.88-fold lower than the DMSO control) (Fig. 6B).

## Discussion

One of the key features of *T. gondii* is the flexible interconversion between tachyzoites and bradyzoites, which allows the parasite to persist in host tissues for long periods. This has significant clinical implications, as tissue cysts that induce long-term persistence are resistant to conventional toxoplasmosis drugs such as atovaquone, pyrimethamine, and sulfadiazine (Araujo et al., 1992; Gormley et al., 1998). Several studies have suggested that the prolonged persistence of *Toxoplasma* in the brain may be associated with neurological diseases, including Parkinson's disease, certain malignancies, and neuropsychiatric disorders such as schizophrenia and epilepsy. However, cyst-enclosed bradyzoites are generally considered harmless in immunocompetent individuals (Miman et al., 2010; Ngo et al., 2017; Tedford and McConkey, 2017). Unfortunately, there are currently no approved treatments that lead to the complete eradication of encysted *Toxoplasma* and cure chronic infection. Consequently, considerable efforts are being made to discover safer and more effective drugs for treating both acute and chronic toxoplasmosis. Among these, small molecule kinase inhibitors (SMKIs), such as HER2, c-MET, and VEGF-2 inhibitors, are promising candidates for targeting both bradyzoites and tachyzoites (Carey et al., 2004; Child et al., 2013; Hua et al., 2023; Kim et al., 2017; Kortagere, 2012; Martinez-Gil et al., 2013; Rutaganira et al., 2017).

Altiratinib was initially identified for its ability to inhibit tumor growth and invasion in glioblastoma and other invasive solid tumors. It has also demonstrated anti-helminthic activity against tachyzoites of *Toxoplasma*, inhibiting multiple targets including receptor tyrosine kinase c-MET, the tyrosine kinase with immunoglobulin and EGF homology domains (TIE2), vascular endothelial growth factor receptor 2 (VEGFR2), and tropomyosin receptor kinase (TRK) (Kwon et al., 2015; Smith et al., 2015; Swale et al., 2022). c-MET is notably active in the cellular microenvironment, where it contributes to tumor progression by enhancing tumor burden, angiogenesis, and invasion through endothelial cell disturbance, as well as recruiting immune cells pathologically (Danilkovitch-Miagkova and Zbar, 2002; Passelli et al., 2022; Soete et al., 1994; Tuck et al., 1996). MET receptors promote their own auto-phosphorylation and amplification through paracrine or autocrine mechanisms, and are associated with signaling pathways related to revascularization and drug resistance, particularly via TIE2, VEGFR, and TRK in cancer treatment (Hashizume et al., 2010; Jahangiri et al., 2013).

In this study, we found that altiratinib was the only small molecule kinase inhibitor (SMKI) that demonstrated anti-toxoplasmic effects against the ME49 strain of *Toxoplasma*. It inhibited intracellular growth in a dose-dependent manner at both early and late stages of infection, while causing minimal damage to the host cells. These findings suggest that altiratinib could be a promising candidate for treating bradyzoite infections.

Apoptosis and autophagy are processes that maintain cellular homeostasis during metabolic stress. However, in pathological conditions such as tumorigenesis, infection, or metabolic dysregulation, kinase molecules can induce aberrant anti-apoptotic or autophagic responses at localized sites in the body (DeLong, 1998; Gupta et al., 1999; Kaur et al., 2021; Kim et al., 2001; Levine and Klionsky, 2004). SMKIs studied in this study appear to cause minimal damage to host cells through apoptosis or autophagy. Specifically, apoptosis was rarely observed with the kinase inhibitors, with the exception of neratinib. Afatinib induced a high autophagy response, while altiratinib resulted in low LC3-II/I expression levels. This suggests that altiratinib may promote cell growth and division, inhibit both apoptosis and autophagy, and regulate mitochondrial biogenesis through increased expression of p-mTOR, p-NF-κB, and Bcl-xL.

Reactive oxygen species (ROS) encompass a broad range of metabolites that can trigger cellular oxidative damage (Jakubczyk et al., 2020). Some pathogens, including *Leishmania* parasites, induce c-MET related signaling regulation to manipulate their survival in host cells and influence ROS release (Carrolo et al., 2003; Leiriao et al., 2005; Passelli et al., 2022; Puthiyakunnon et al., 2017). Pathological ROS production from the *Toxoplasma*-infected hosts is particularly lethal to neuronal tissues, including neural retina and retinal pigment epithelial (RPE) cells (Choi et al., 2020; Sun et al., 2019; Woods et al., 1998). In this study, altiratinib demonstrated the ability to inhibit bradyzoite reactivation while preserving the viability of host ARPE-19 cells by partially downregulating ROS generation. The observation of the lowest ROS production with altiratinib treatment, compared to the control group, pyrimethamine, or ACTB-1003, further supports the potential of altiratinib as a strong candidate for treating bradyzoite infections of *Toxoplasma*.

*Toxoplasma* infection spreads through the bloodstream and infects many tissues, including skeletal and cardiac muscles, the central nervous system (CNS), and the eyes (Montoya and Liesenfeld, 2004). Retinochoroiditis caused by *Toxoplasma* occurs at a rate of 20–80% due to congenital infection, and severe toxoplasmosis of the CNS develops in immunocompromised individuals, including pregnant women (Yu et al., 2023). The efficacy of drugs in penetrating the blood-brain barrier (BBB) and the retinal blood barrier (RBB) is crucial for treating severe toxoplasmosis. Oral altiratinib has demonstrated the ability to cross the BBB in experimental applications, indicating its potential effectiveness in targeting *Toxoplasma* infections within the CNS (Piao et al., 2016; Smith et al., 2015). Acute *Toxoplasma* infections are typically self-limiting in immunocompetent individuals. However, dormant bradyzoites that reactivate in immunocompromised patients are of clinical significance, as there are currently no approved therapeutic strategies to effectively treat these reactivated infections (Cerutti et al., 2020).

In summary, altiratinib demonstrated the most effective anti-*Toxoplasma gondii* activity at the lowest concentration against the ME49 strain, comparable to its effect on the RH strain. This efficacy was evident at both early and late stages of infection. Furthermore, the Apoptosis/Necrosis Assay and ROS assay confirmed that altiratinib caused no significant adverse effects on normal cells.

This study provides substantial evidence that altiratinib is a promising candidate for treating toxoplasmosis, particularly in targeting bradyzoite reactivation in vitro. Its effectiveness and minimal adverse effects on host cells suggest that altiratinib could play a key role in the development of therapeutic strategies against chronic *Toxoplasma* infections.

## Supplementary Information

Fig. S1. The anti-Toxoplasma activity of TKIs against the RH strain. Based on these findings, the concentrations of afatinib and neratinib (inhibitors of HER2/HER4) were set at 5 μM for the current study. Kim, Y. H., Bhatt, L., Ahn, H. J., Yang, Z., Lee, W. K., and Nam, H. W. 2017. Suppressors for human epidermal growth factor receptor 2/4 (her2/4): A new family of anti-toxoplasmic agents in arpe-19 cells. Korean J Parasitol. 55, 491-503.

Fig. S2. The anti-Toxoplasma activity of ACTB-1003, regorafenib, altiratinib and foretinib against the RH strain. Based on these findings against the RH strain, further experiments against the ME49 strain were performed to set the SMKI concentrations for the current study.
